# Peatland Pond Microbiome and Biogeochemical Responses to Solar Radiation Extremes in a High-Altitude Wetland, Salar de Huasco, Chile

**DOI:** 10.3390/microorganisms13091990

**Published:** 2025-08-26

**Authors:** Yoanna Eissler, Alfredo Yanez-Montalvo, Paula S. M. Celis-Plá, Marcela Cornejo-D’Ottone, Andrés Trabal, Cristina Dorador, Claudia Piccini, Luisa I. Falcón, Carlos Romero, Polette Aguilar-Muñoz, Verónica Molina

**Affiliations:** 1 Laboratorio de Virología, Centro de Neurobiología y Fisiopatología Integrativa, Instituto de Química, Facultad de Ciencias, Universidad de Valparaíso, Gran Bretaña 1111, Playa Ancha, Valparaíso 2360102, Chile; yoanna.eissler@uv.cl; 2 Instituto de Ecología, Unidad Mérida, UNAM, Ucú 97357, Yucatán, Mexico; yanez.alfredo@uabc.edu.mx (A.Y.-M.); falcon@ecologia.unam.mx (L.I.F.); 3 Laboratorio de Ecología y Conservación, Facultad de Ciencias, Universidad Autónoma de Baja California, Carretera Transpeninsular 3917, Fraccionamiento Playitas, Ensenada 22860, Baja California, Mexico; 4 Laboratory of Aquatic Environmental Research, Universidad de Playa Ancha, Valparaíso 2360004, Chile; paulacelispla@upla.cl (P.S.M.C.-P.); andres.trabal@gmail.com (A.T.); 5 Departamento de Ciencias y Geografía, Facultad de Ciencias Naturales y Exactas, Universidad de Playa Ancha, Leopoldo Carvallo 270, Playa Ancha, Valparaíso 2340018, Chile; cromero@upla.cl (C.R.); polette.aguilar@upla.cl (P.A.-M.); 6 HUB Ambiental UPLA, Universidad de Playa Ancha, Leopoldo Carvallo 207, Playa Ancha, Valparaíso 2340018, Chile; 7 Escuela de Ciencias del Mar and Núcleo Milenio para el estudio de la Desoxigenación del Océano Pacífico Sur oriental (DEOXS), Pontificia Universidad Católica de Valparaíso, Avenida Universidad 330, Valparaíso 2360007, Chile; marcela.cornejo@pucv.cl; 8 Facultad de Ingeniería, Negocios y Ciencias Agroambientales, Universidad Viña del Mar, Diego Portales 90, Viña del Mar 2580022, Chile; 9 Laboratorio de Complejidad Microbiana y Ecología Funcional, Instituto de Antofagasta, Departamento de Biotecnología, Facultad de Ciencias del Mar y Recursos Biológicos, Universidad de Antofagasta, Avenida Universidad de Antofagasta s/n, Antofagasta 1240000, Chile; cristina.dorador@uantof.cl; 10 Centre for Biotechnology and Bioengineering, Universidad de Chile, Beaucheff 851 (Piso 7), Santiago 8320000, Chile; 11 Departamento de Microbiología, Instituto de Investigaciones Biológicas Clemente Estable IIBCE, Av. Italia 3318, Montevideo 11600, Uruguay; cpiccini@iibce.edu.uy; 12 Laboratorio de Teledetección Ambiental, Departamento de Ciencias Geográficas, Facultad de Ciencias Naturales y Exactas, Universidad de Playa Ancha. Avenida Leopoldo Carvallo 270, Playa Ancha, Valparaíso 2360002, Chile; 13 Centro de Investigación Oceanográfica COPAS COASTAL, Universidad de Concepción, Concepción 4070409, Chile

**Keywords:** chlorophyll *a*, UV radiation, greenhouse gases, nutrient rates, microbial community, diel patterns, high-altitude wetland, Salar de Huasco

## Abstract

High-altitude wetland holds unique peatland ponds subjected to extreme diel environmental condition changes. Herein, we evaluate the response of photoautotrophic and nitrification activities and compare it with bacteria and archaea composition shifts in sediment and water changes during key hours of the day. Results indicate the presence of photo-inhibition, including ammonia oxidizers, but a high recovery of photosynthetic activities in the microbial mat and of potential specific functional groups towards the afternoon. The microbial community was composed of 45 phyla, mainly proteobacteria from Alpha-, Delta-, and Gammaproteobacteria and Bacteroidota in the water and sediments, and these later groups were notoriously enriched during the afternoon. The microbial community composition changes were associated with chlorophyll *a*, nutrients, and greenhouse gases reservoir variability, including methane potential release towards the atmosphere at hours of high radiation. Peatland pond microbial communities and their biogeochemical contribution change in a complex interplay coupled by time to environmental conditions predominantly driven by the extreme solar radiation.

## 1. Introduction

Solar radiation is the main driver of life on earth; phototrophic organisms harvest light for growth, fueling biogeochemical cycles and trophic webs [1]. During each daily cycle, photoautotrophs must cope with natural irradiance fluctuation, including light stress at hours of high solar radiation directly inhibiting their metabolism due to saturation of the light centers of the reaction [2] or indirectly due to altered water physicochemical properties [3]. In addition, solar radiation can be harmful to other key microbial communities known for their role in nutrient and greenhouse gas recycling, such as nitrifying microorganisms that oxidize ammonia to nitrate via nitrite and produce N_2_O as a side product [4]. Photo-inhibition associated with photosynthetically active radiation (PAR) and ultraviolet radiation (UVR) is a well-documented factor that affects nitrifying bacteria and archaea [5]. Indirectly, UVR could relieve aquatic microbial communities’ top-down control through viruses since viral particles could be destroyed by high UVR (reviewed by [1]). Photochemical reactions such as photo ammonification could enhance microbial aquatic activities, enhancing substrate bioavailability [6,7]. In addition, phototrophs and other microorganisms could overcome the stress caused by solar radiation, recovering their metabolic capacity based on adaptive molecular mechanisms to tolerate stress, e.g., osmoprotectant production in response to UVR in *Rhodobacter* sp. [8], a bacteria isolated in high-altitude wetlands.

High-altitude wetlands are considered a hotspot of microbial life [9], where this and other organisms are exposed to the effects of extreme physicochemical gradients. Situated at elevations > 2500 m above sea level, these ecosystems are exposed daily to extreme changes associated with solar radiation, temperature shifts, and wind velocity, where life develops in diverse water bodies, i.e., freshwater spring, streams, and shallow saline lagoons [10,11]. In particular, peatland constitutes a significant portion of the wetlands landscape and is characterized by cushion plants locally termed “bofedales”, which surround water ponds in the Andean puna shared with Bolivia and Argentina [10]. In general, peatland areas are significant carbon storage zones, representing up to 2.84% of the global total carbon storage land area [12]. Few studies have focused on the biogeochemical role of peatland sites associated with the high-altitude wetlands of Chile despite their potential contribution of organic matter recycling in the desert ecosystem of the Andes (altiplano), where semipermanent pond systems have developed [13]. These areas are carbon and water reservoirs currently threatened by drought due to climate change and human intervention, especially due to water use from mining and other activities [12].

Salar de Huasco is currently a National Park with a low anthropogenic impact and an ideal natural laboratory to identify potentially synergistic responses to the poly-extreme conditions [7,12,13]. This high-altitude wetland, as others from the Andes, is characterized by a main lake, streams, and peatland ponds with variable physical and chemical conditions, i.e., salinity, nutrient, dissolved organic carbon, and greenhouse gases content, which are associated with changes in microbial community beta diversity [14], including virus-to-prokaryote ratio [15] and specific functional groups composition shifts, such as nitrifying bacteria and archaea [16]. Peatland ponds are supported by groundwater springs in Salar de Huasco and cover a large surface area of the wetland, reaching a similar magnitude as the main lake surface: c.a. 250 ha [13]. Ponds hold conspicuous microbial traits such as high diversity in the water and sediments, including the development of biofilms and microbial mats [17,18]. Microbial activities in ponds contribute to high carbon and nitrogen recycling in the wetland [13,19]. Microbial mats and sediments carry a significant primary productivity in this ecosystem [20] and could generate reducing potential for oxidative processes, like ammonia, sulfur, and methane oxidation [13]. So far, little is known about the mat biological responses and changes in a daily cycle and how this performance could be associated with other biogeochemical conditions in ponds. In this study, we evaluate the diel responses of microbial communities and their potential biogeochemical functions under extreme environmental changes throughout the day and explore the potential tolerance of key processes such as photosynthetic performance of microbial mats and their potential association with microbial community composition and nitrifying community responses in a pond representing the peatland area of Salar de Huasco.

## 2. Materials and Methods

### 2.1. Study Site, In Situ Analyses, and Sample Collection

The study area was visited during September 2019 (16 and 17th) at the pond area associated with the sampling site (H3, −20.28° S, −68.88° W; see Figure S1). This sampling area has been already surveyed for microbial diversity and other processes (e.g., [11,18]).

**Figure 1:**
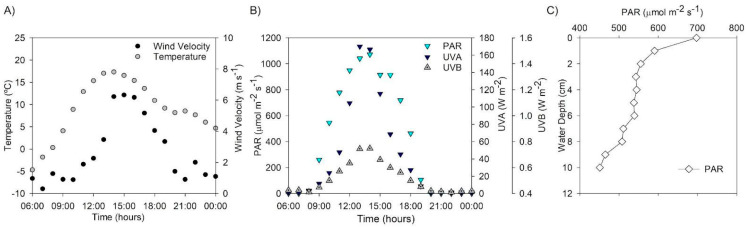
Environmental variables along the experimental time: (**A**) air temperature and wind velocity variability; (**B**) solar radiation including PAR, UVA, and UVB daily changes; (**C**) light attenuation in the pond water.

Meteorological parameters such as air temperature and wind speed were obtained from the station situated at −20.26° S, −68.87° W and are publicly available (https://www.ceazamet.cl) and shown in Figure 1A. The photosynthetically active radiation (PAR; ʎ = 400–700 nm), and ultraviolet A radiation (UVA; 320–400 nm) were measured in the air using QSO-SUN 2.5V and USB-SU 100 cosine-corrected sensors (Apogee Instruments, Logan, UT, USA) (Figure 1B). PAR diffuse attenuation coefficient (K_dPAR_) in the water was calculated measuring PAR radiation just below the water surface and every 0.1 m depth in the water column with a spherical PAR sensor (US-SQS, Walz GmbH, Effeltrich, Germany) (Figure 1C). The attenuation coefficient was estimated using the Beer–Lambert law equation:

E_d_(z) = E_d_(0)·e^−Kd·z^ (1)

E_d_(z) is the irradiance (PAR) measurement at depth z, E_d_(0) is the irradiance measurement when the sensor is just under the water surface, K_dPAR_ is the diffuse attenuation coefficient, and z is the depth.

The study was centered in a pond with approximately 15–20 cm depth characterized by the presence of an unconsolidated microbial mat at the sediment surface (Figure S2c). Physicochemical conditions of the water were determined in situ using a portable multiparameter (HANNA instrument model HI9829, Woonsocket, RI, USA) (Figure S3). Water samples for nutrient determinations were collected directly using sterile syringe and filtered through a GF/F filter (Whatman, Maidstone, UK), and the filtrates were stored in rinsed Nalgene flasks (125 mL, in triplicate). Water for chlorophyll *a* (500 mL) analyses was sampled and filtered onto GF/F filters using a vacuum pump system and filters and was stored frozen until laboratory analyses. These samples were frozen in the field and stored at −20 °C until analysis in the lab. Water samples for greenhouse gases (GHG) were collected into vials (20 mL, in triplicate), avoiding bubbles, and poisoned with 50 µL of saturated mercuric chloride. These samples were stored at room temperature under dark conditions until analyzed. Figures S2 and S3 illustrate the area of sampling and in situ procedures.

Water and sediment were sampled to determine microbial community composition for subsequent metabarcode (iTag) 16S rRNA gene sequencing. Surface sediment samples (~500 µL) (corresponding to microbial mats) were collected using a sterile spatula and stored in cryovials with RNAlater solution (Ambion, Life Technologies, Carlsbad, CA, USA). The water (~1 L) was filtered to collect planktonic microbial communities using a peristaltic pump and Sterivex filter (SVGP0150, Merck Millipore, Burlington, MA, USA) for DNA analyses, and the samples were filled with RNAlater solution, a nucleic acid stabilizer used for both DNA and RNA [21]. The filters were then frozen in liquid nitrogen in the field and stored at −80 °C until analysis.

### 2.2. Nutrients and GHG Analyses

Nutrients, silicic acid, nitrate, nitrite, and phosphate were analyzed using standard colorimetric methods with an automatic nutrient analyzer following [22]. For GHG determinations, the water was displaced using ultrapure helium gas to generate a headspace that was sampled after equilibrium to determine GHG [23]. GHG was analyzed using a gas chromatograph (GC-2014 Greenhouse, Shimadzu, Kyoto, Japan) using an electron capture detector (ECD) for N_2_O, i.e., a methanizer for CO_2_ conversion to CH_4_, and a flame ionization detector (FID) for CH_4_. A three-point calibration curve with helium, air, and a standard of 600, 5, and 1 ppm for CO_2_, CH_4_, and N_2_O, respectively, was used for GHG concentration determination (Scotty gas mixture; Air Liquid Co., Paris, France).

### 2.3. Experiments Carried out to Evaluate GHG Exchange and Nutrient Recycling in the Pond Area

To evaluate GHG exchange between the water and atmosphere, a floating flux gas chamber was installed on the water pond surface during the afternoon. Air samples were collected during the afternoon every 20 min (16:00–17:20 h) to identify short GHG changes before sunset, based on a previous study [13]. Sampling was carried out through a septum and then filled into vials containing a salt-saturated water solution to avoid gas leak. GHG analyses were carried out as described above for discrete samples, without the need of a headspace step.

Net changes of nutrients were performed using the experimental design based on short endpoint incubations and four treatments with inhibitors of nitrifying metabolisms with water collected at dawn (without the effect of solar radiation) and in the afternoon. Samples were distributed into Nalgene (125 mL), with duplicates for each of the four treatments and one set as control (without inhibitors). Samples were incubated under dark and temperature-controlled conditions during 5–6 h. To stop the experiment, the water was filtered using a sterile syringe through a GF/F filter (Whatman), and the filtrate was collected in clean Nalgene flasks (125 mL) and frozen −20 °C for nutrient analyses as described above. The rationale behind this experiment was to determine the potential effect of photo-inhibition of the different steps of nitrification process (ammonia and nitrite oxidation) and the potential contribution of archaeal ammonia oxidation using a combination of inhibitors that were selected based on our previous experience in the study area [16]. The nitrogen metabolism inhibitors used were allylthiourea (ATU), which added to a final concentration of 84 mM inhibits ammonia-oxidizing bacteria and partially archaea [24,25]; N1-guanyl-1, 7-diaminoheptane (GC7) at a final concentration of 0.4 μM, which is an archaeal antibiotic [26] and previously used to evaluate archaeal versus bacterial ammonia oxidation [16,27]; and sodium azide (Azide) added to a final concentration of 34 μM used to inhibit nitrite oxidation and denitrification [24].

Endpoint estimations were determined by subtracting the initial nutrient concentration from the final nutrient concentration and dividing it by the total water incubation hours. The following equations were used to estimate the potential of nitrification and other processes in the net accumulation of the control nitrite rates of change *R* (μM h^−1^):

Ammonium oxidizing bacteria contribution:

*R*_*AOB*_NO_2_^−^ = *R*_*Azide*+*GC7*_NO_2_^−^ − *R*_*CONTROL*_NO_2_^−^ (2)

Ammonium oxidizing archaea contribution:

*R*_*AOA*_NO_2_^−^ = *R*_*Azide*+*ATU*_NO_2_^−^ − *R*_*CONTROL*_NO_2_^−^ (3)

Other processes contribution to the production or consumption of nitrite (+ or − rate):

*R*_*Other Processes*_NO_2_^−^ = *R*_*Azide*_NO_2_^−^ − *R*_*CONTROL*_NO_2_^−^ (4)

### 2.4. Photosynthetic Activity, In Vivo Chlorophyll a Fluorescence Measurement, and Parameters

The in vivo chlorophyll *a* fluorescence was determined by pulse amplitude modulated fluorometers (Junior-PAM and Mini-PAM) following [2,28].

The in situ measurement was performed in the pond sediments where microbial mats develop. The fiber optics together with a spherical PAR quantum sensor US-SQS (Walz GmbH, Effeltrich, Germany) were submerged in the water column and placed in the sediment to the measurement of the Yield II and *F_v_*/*F_m_*. Blue and red light-emitting diodes (LED; 460 nm, 655 nm) in the control box were powered by PC via a USB interface (Figure S2) and measured actinic light and saturating pulses. WinControl-3 software 3.2 was used for data acquisition and recording.

The incident photosynthetically active irradiance (E-PAR; ʎ: 400–700 nm) and the effective quantum yield (Yield II or *ΔF*/*F_m_′*) were measured every 2 h during the light period, and the *ΔF*/*F_m_′* was calculated according to [29]:

*ΔF*/*F_m_′* = *(F_m_′ − F)*/*F_m_′* (5)

where *Fm′* is the maximal fluorescence induced with a saturating blue-light pulse and *F* the current steady-state fluorescence in light-adapted photoautotrophic communities.

The relative electron transport rate through PSII (rETR; r.u.) was determined as follows:

*rETR* in situ = *ΔF/F_m_′ × E-PAR* (6)

Maximum quantum yield of PSII (*F_v_/F_m_*) is an indicator of physiological status of the phototrophic communities and photo-inhibition [29]. *F_o_* (basal fluorescence from fully oxidized reaction centers of PSII) and *F_m_* (maximal fluorescence from fully reduced PSII reaction center) were determined in darkness to obtain the maximal quantum yield (*F_v_/F_m_*), with being *F_v_* the difference between *F_m_* and *F_o_* [2,29].

### 2.5. Total DNA Extraction and 16S rDNA Amplification

Nucleic acid extraction from the sediments was performed by using the PowerSoil™ DNA Isolation Kit (MoBio Laboratories, Solana Beach, CA, USA) in accordance with the manufacturer’s specifications. All DNA extracts were resuspended in 50 µL of nuclease-free water, and their concentration was determined by fluorescence using Qubit Fluorometer (Thermo Fisher Scientific, Waltham, MA, USA). The DNA extracts were stored at −80 °C until further analysis. 16S rDNA gene amplification was performed using 27F(5′-AGRGTTYGATYMTGGCTCAG-3′)/519R (5′-GWATTACCGCGGCKGCTG-3′) primers and a PCR reaction consisting of 95 °C for 5 min followed by 35 cycles of 95 °C for 45 s, 56 °C for 45 s, and 72 °C for 45 s and a final elongation step of 5 min at 72 °C using GoTaq^®^ DNA Polymerase (Promega Corporation, Madison, WI, USA). Amplification results were verified using electrophoresis for 45 min at 70 volts in an agarose gel (1.5%). DNA extracts were sequenced using the Illumina Miseq sequencing platform at Mr. DNA laboratory (Shallowater, TX, USA), using in-house primers 515F (GTGYCAGCMGCCGCGGTAA) [30] and 806R (GGACTACNVGGGTWTCTAAT) [31] for the V4 region of the 16S rDNA gene. Sequences were deposited in the European Nucleotide Archive (ENA) under the project accession PRJEB79584 (Accession number ERR13620671–ERR13620681).

### 2.6. Bioinformatic Processing of Illumina 16S rDNA Sequences and Biostatistical Analysis

Raw sequences were imported and processed in QIIME2 (QIIME 2, v.2018.6) [32], and sequences were clustered into amplicon sequence variants (ASVs) and were assigned taxonomy using the SILVA database (release 138–99% OTUs, 515–806 region) [33]. Based on quality plots, forward and reverse reads were truncated at their 3′ end at the 200 sequencing positions, respectively. Chimeric sequences were removed using the dada2 pipeline [34]. ASVs were grouped to 100% sequence similarity. Sequences were aligned using MAFFT [35], and rooted trees were constructed using FastTree for analysis of phylogenetic diversity [36].

Subsequently, all sequence data were analyzed in the R statistical environment (version 3.6.2), with Phyloseq R [37], ggplot2 (v 2.1.0) [38], and vegan packages (v2.3–5) [39], while ampvis2 [40] and pheatmap [41] were used for data visualization and statistical testing. ASVs representing less than 1000 sequences across the dataset and all singletons and mitochondrial sequence reads were eliminated using customized R scripts. Chloroplasts were used to identify eukaryotic phototrophs’ contribution.

The bacterial and archaeal genetic composition of the pond was described along the sampled water and sediments, using sequences representing > 2 % of relative abundance at the phylum level. Biological diversity was used to determine the similarity in the community structure of samples and was explored using the alpha diversity metrics (Shannon and Simpson and observed ASVs). Venn diagrams were constructed using the software Venny [42] to compare the microbial composition among the pond aquatic compartments (sediments versus water). To visually inspect the variation of the microbial community in the pond, a heatmap was generated by pheatmap 1.0.7.

Functional groups associated with nitrification assemblages and photosynthesizers were analyzed based on the identification of well-known microbial communities in our sequencing database by a bibliographic analysis following [43]. In addition, functional predictions based on taxonomy were performed using the *trans_func* function of the microeco package (v 1.8.0) (10.1093/femsec/fiaa255), specifying the FAPROTAX database (10.1126/science.aaf4507) for prokaryotes.

### 2.7. Statistical Analysis

Differences in prokaryotic community composition between pond water and sediment were assessed using canonical analysis of principal components [44,45] and was tested using the CAPdiscrim function in the package BiodiversityR [46]. A linear discriminant analysis (LDA) was carried out to identify microbial ASV shifts comparing morning versus afternoon microbiomes in the water and sediment results having a threshold LDA score > 2 [46]. All the indices for alpha diversity were compared using tests of Mann–Whitney–Wilcoxon. For beta-diversity, PERMANOVA analyses were performed using the Adonis function of the vegan package, based on the weighted UniFrac matrix. All correlations and tests with *p*-value < 0.05 were considered significant after 1000 permutations. Spearman correlation coefficients were calculated and tested between all measured dependent variables using Statistica software version 7.0. Following the assessment of normality (Shapiro test) and homoscedasticity (Levene’s test), biogeochemical differences between morning and afternoon were evaluated using a one-way ANOVA (*aov* function, stats package (v 4.2.2)) [47]. When assumptions were not met, a non-parametric Kruskal–Wallis test (*kruskal.test* function, stats package) was conducted.

## 3. Results

### 3.1. Environmental, Biogeochemical, and Light Changes

The environmental conditions in the study area were characterized by extreme changes during the day (Figure 1). The air temperature varied from −5 °C before sunrise up to 18 °C at noon and early afternoon (13:00 h–15:00 h), and wind velocity presented a steep increment from 1 to 6 m s^−1^ at noon (Figure 1A). The abiotic variables were associated with solar radiation changes, with a peak of the irradiance of 1000–1150 µmol m^−2^ s^−1^ for PAR, 160–170 W m^−2^ for UVA, and 58 W m^−2^ for UVB during the experimental period (Figure 1B). The K_dPAR_ in the pond’s water during the experimental period was 0.32 cm^−1^. PAR attenuation decreased by 20% in less than 2 cm of water depth (Figure 1C).

**Figure 2:**
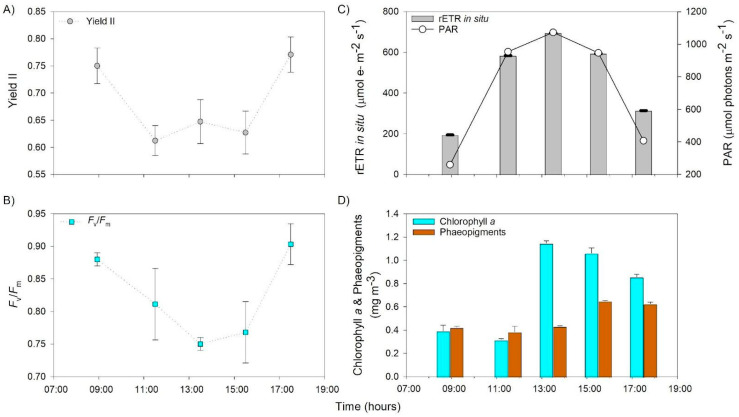
Photosynthetic performance during daily cycle experiment, (**A**) effective quantum yield or Yield II, (**B**) maximal quantum yield as *F_v_/F_m_*, (**C**) in situ relative electron transport rate as in situ rETR and PAR, and (**D**) chlorophyll *a* and phaeopigments in the water.

The effective quantum yield (Yield II) decreased with the higher irradiances between 800–1000 mmol photons m^−2^ s^−1^, reaching the minimal values in 11:00 and 15:00 h with around 0.6 values (Figure 2A). The maximal quantum yield or *F_v_/F_m_* varied significantly depending on time (*p* < 0.05) (Figure 2B). *F_v_/F_m_* was high in the samples collected in the morning and late afternoon, showing the lower values (0.75) at midday (Figure 2B).

The in situ relative electron transport rate (rETR in situ) and irradiance showed a similar behavior during the day. The rETR in situ showed maximal values at 13:00 h, around 700 µmol m^−2^s^−1^ (Figure 2C), and at the same time, an increase in Chl*a* was observed in the pond, showing a reduction during the afternoon in coincidence with phaeopigment increment (Figure 2D).

The physical and chemical conditions in the pond water were characterized by a conductivity of 2176–3692 µS cm^−1^ and alkaline conditions (pH of 9.08–9.3 units) (Table S1).

**Figure 3:**
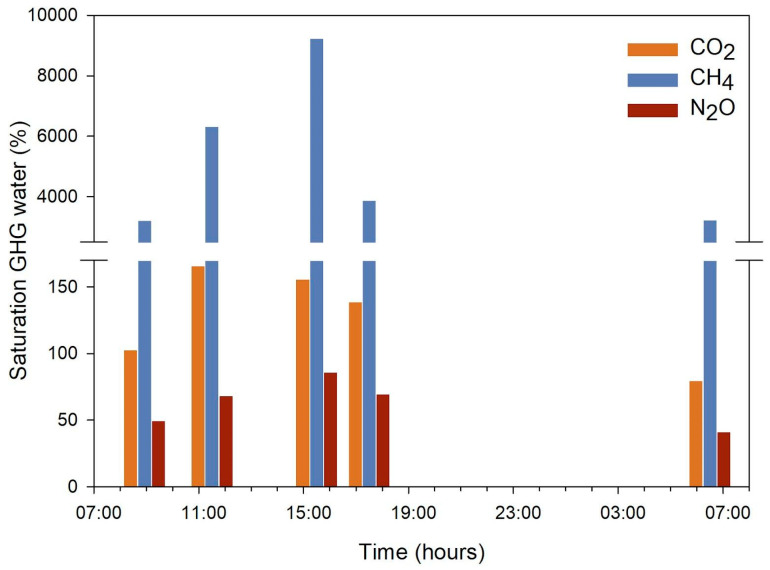
Dissolved greenhouse gases saturation in the water of the pond during the sampling hours.

Dissolved greenhouse gases CO_2_, CH_4_, and N_2_O were on average 23.4 µM, 163.1 nM, and 7.3 nM, respectively, showing a variable concentration in the pond water GHG (Table S1). However, only CH_4_ and CO_2_ were supersaturated in the water the first day during all the sampling hours, especially at hours of high radiation 11:30 and 15:30 h (Figure 3). In contrast, N_2_O was subsaturated. Gas flux chamber results recorded during the afternoon (15:50–17:20 h; see Figure S4) agreed with the dissolved GHG concentration, showing a net CH_4_ accumulation, a more variable CO_2_ with a decreasing tendency, and an invariable N_2_O concentration change through time.

**Figure 4:**
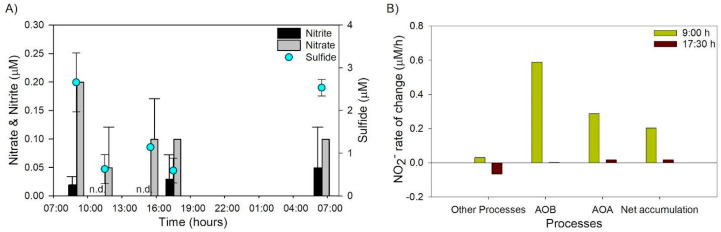
(**A**) Nitrate, nitrite, and sulfide changes in the daily cycle and (**B**) the nitrification potential evaluated through nitrite rates of change in the morning and afternoon.

Dissolved nutrients were low but variable through the day, showing higher concentration at low radiation hours (morning and late afternoon), characterized by detectable nitrite and nitrate concentrations ≥ 0.05 µM (Figure 4A). Phosphate presented a slight concentration increment in the afternoon (Table S1). In the morning of both days sampled, higher concentrations of silicic acid (Table S1) and sulfide were registered in the pond (Figure 4A).

The comparison between morning and afternoon concentrations indicated that only CH_4_ presented a tendency to accumulate towards the afternoon, whereas the opposite was observed for the rest of the gases and nutrients, characterized by a significant difference based on ANOVA for N_2_O, H_2_S, phosphate, and silicic acid (Figure S5).

The result of nutrient rates of change based on end-point experiments indicated a higher accumulation of nitrite compared with the other nutrients analyzed, specifically during the morning, with rates ranging between 0.20 and 0.79 μM h^−1^ (Table S2). The comparison between the nitrite rates of change obtained from the different treatments (see Equations (2) and (3) in the Section 2) showed differences consistent with activity from both ammonia-oxidizing bacteria (AOB) and archaea (AOA) (Figure 4B). Rates measured in the morning were higher than those in the afternoon. In addition, other processes contributing to both nitrite consumption and accumulation were evident in our experiments, as indicated by nutrient endpoint rates determined in the control and azide treatments (Table S2, Figure 4B). Nitrate and phosphate rates of change shifted from high net consumption during the morning to accumulation during the afternoon (Table S2).

Spearman rank analyses revealed significant (*p* < 0.05) correlations between solar radiation quality and environmental variables, including positive correlations between PAR and both UVA and UVB and negative correlations such as UVA/nitrite, UVB/silicic acid, and CO_2_/phaeopigments (Table S3). Photosynthetic performance parameters also showed significant associations with environmental variables; for example, Yield II and *F_v_/F_m_* were positively correlated with nitrite, whereas *F_v_/F_m_* was negatively correlated with UVB and methane (Table S3). Furthermore, picoplankton and viral-like particle (VLP) abundances estimated during the same sampling [15] were significantly correlated with solar radiation and photosynthetic responses, displaying positive correlations with Yield II and nitrite and negative correlations with PAR and UVA (Table S3).

### 3.2. Microbial Composition in the Water and Sediments of the Pond and Its Variability During Morning and Afternoon

Microbial communities were rich and diverse, reaching up to 1719 different ASVs and 6.8 H’, especially in sediments where higher diversity indexes were found (Table S4). Benthic microorganisms differed significantly from their planktonic counterpart (pairwise comparisons using Wilcoxon rank sum test; *p* = 0.01) and clearly were observed in the PCoA analyses (Figure S6).

**Figure 5:**
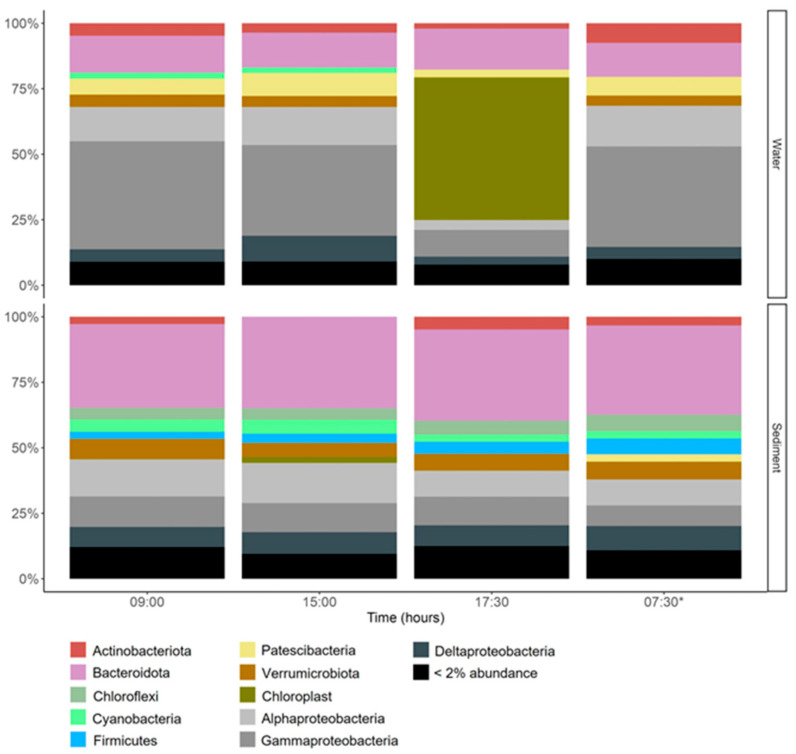
Microbial community composition shifts in the water and sediments. 07:30 *: second-day measurements.

The microbial community composition was represented by 45 phyla mainly associated with Proteobacteria phylum (currently Pseudomonadota) of the classes (Alpha- and Gammaproteobacteria) and the phylum Deltaproteobacteria in the water and Bacteroidota in the sediments (Figure 5). Shifts during different sampling times were observed; for example, planktonic Gammaproteobacteria, Deltaproteobacteria, and Patescibacteria were characterized by a decrease in the afternoon (17:30 h), whereas an increase was observed for Bacteroidota (Figure 5). These changes were associated with specific genera (Figure S7), such as Hydrogenophaga and Polynucleobacter (Gammaproteobacteria), *C*. Aquiluna (Actinobacteriota), Peredibacter and Bdellovibrio (Deltaproteobacteria), and Algoriphagus and Flavobacterium (Bacteroidota). Chloroplasts presented a significant contribution to the planktonic microbial community in the afternoon (Figure 5). In this case, a group of eight ASVs were detected (Figure S7). In the sediments, Bacteroidota, Chloroflexi, and Verrucomicrobiota, among others, increased in comparison with water, and Alphaproteobacteria decreased in the afternoon and the next day at sunrise (Figure 5).

Microbial communities differed significantly between morning and afternoon. Discriminative analysis identified taxa with predominant shifts in both water and sediment, mainly affiliated with Gammaproteobacteria, with a greater number of shifts observed in sediments (Figure S8).

### 3.3. Functional Shifts of Specific Microorganisms

Functional characterization of microbial communities in water and sediments indicated a potential specialization in the studied shallow peatland pond. Predictions based on taxonomic profiles revealed shifts in key biogeochemical cycles between morning and afternoon samplings (Figures S9 and S10). In water, morning samples showed enrichment in photoheterotrophy, nitrogen fixation, sulfur compound respiration, and fermentation, whereas afternoon samples exhibited higher variability in predicted functions (Figure S9). In sediments, a greater proportion of anaerobic processes, including sulfur and nitrogen cycling and anoxygenic photosynthesis, was observed, particularly in the 17:30 h community (Figure S10).

**Figure 6:**
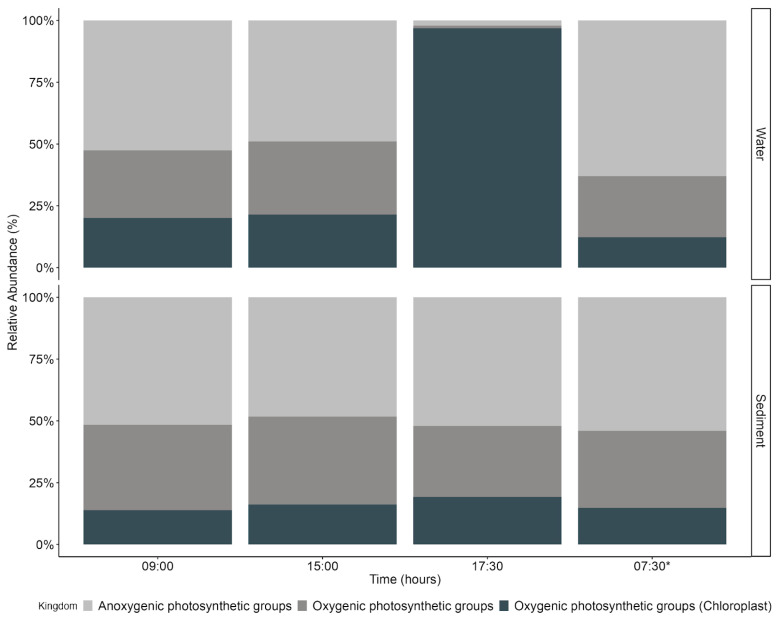
Anoxygenic and oxygenic photosynthetic groups in the water and sediments. 07:30 *: second-day measurements.

The specific functional group analyses performed for photosynthetic metabolism indicate that anoxygenic photosynthetic groups were more abundant in sediment than in water, and in both the sediment and water, oxygenic photosynthetic groups increased in the afternoon, especially with a peak in the water (17:30 h), and decreased at dawn the next day of sampling (Table S5, Figure 6). This result coincides with the recovery of photosynthetic performance measured in the sediment mat through *F_v_/F_m_* (Figure 2).

**Figure 7:**
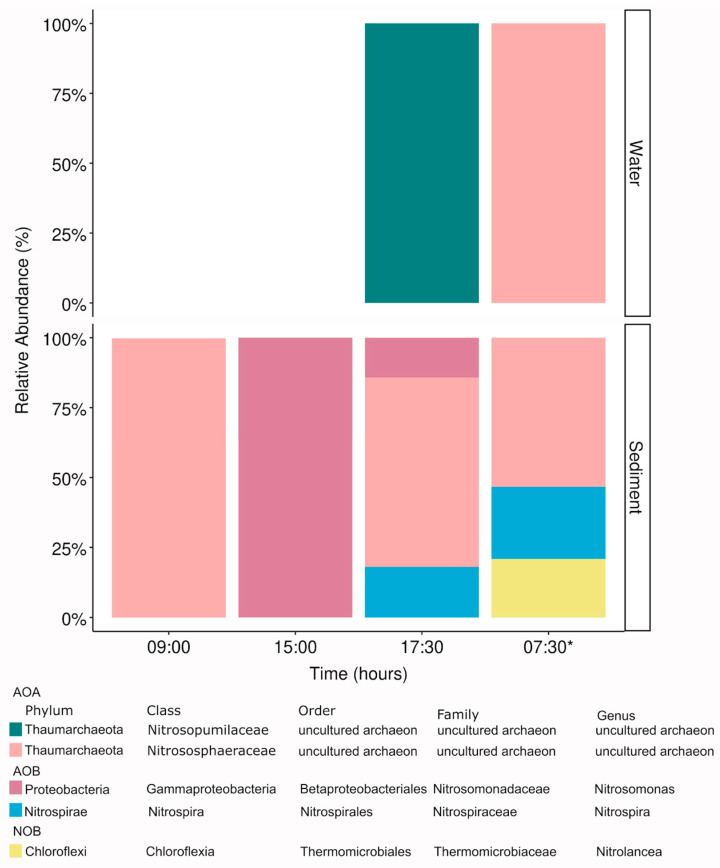
Nitrifying assemblages detected in the water and sediments.

In general, in the afternoon, a higher diversity of functional groups associated with nitrifying assemblages was detected both in the water and sediment (Figure 7). Nitrosopumilaceae and Nitrososphaeraceae were the dominant families showing a higher variability in the water (Figure 7), including other archaea taxa associated with methanogenesis (Figures S11 and S12). In the water, the enrichment of AOA in the microbial community in the afternoon coincided with the detection of water ammonia oxidation rates by archaea but not of bacteria (Figure 4B). Whereas in the sediment, nitrifying bacteria groups of the genera *Nitrosomonas*, *Nitrospira*, and *Nitrolancea* were also present (Figure 7). Specifically, *Nitrosomonas* completely dominated at 15:00 h to disappear at 7:30 h the next day, and *Nitrolancea* nitrite oxidizer appeared at the same time.

The relevance of taxa associated with other functions, such as organic matter remineralization, including sulfur metabolisms like Deltaproteobacteria, coincided with a reduction in photosynthetic performance based on *F_v_/F_m_* (Figure 2) and the accumulation of reduced compounds such as sulfide (Figure 4A).

## 4. Discussion

### 4.1. Interaction Between Extreme Environmental Conditions and Biogeochemistry of a High-Altitude Pond

During our study, substantial fluctuations in irradiance, air temperatures, and wind velocity indicated the prevalence of poly-extreme conditions, as previously reported for Salar de Huasco [12,13]. Photo-inhibition was directly measured in benthic phototrophic communities via PAM fluorometry (Figure 2), whereas inhibition of planktonic nitrifiers was inferred from the decline in nitrification rates (Figure 4B) and the temporal disappearance of potentially sensitive specific taxa such as Nitrosomonas and nitrite-oxidizing bacteria in the sediment microbial mat (Figure 7). This distinction highlights the differing sensitivities and ecological roles of benthic versus planktonic microbial communities under solar radiation stress [5,8]. During peak irradiance periods, photosynthetic parameters, specifically effective quantum yield (Yield II) and maximal quantum yield (*F_v_/F_m_*), display notable declines, indicating photo-inhibition effects around noon and early afternoon of the sediment microbial community (Figure 2). Concurrently, in situ relative electron transport rates (rETR in situ) showed peak activity at midday, followed by a decline, correlating with changes in chlorophyll *a* and phaeopigment concentrations. The diel variations in photosynthetic performance observed in this study align with previous research highlighting the impact of light intensity on aquatic photosynthetic organisms [48]. Similar patterns of decreased *F_v_/F_m_* values during midday due to photo-inhibition have been reported in various aquatic environments [48]. Despite photo-inhibition, the photosynthetic microbial community in the sediments of the study area presented a high recovery in the afternoon (Figure 2), potentially due to the plethora of stress tolerance and adaptation strategies described to overcome stress (e.g., [49]) that enable them to contribute to the high primary productivity registered in this study area [20]. In addition, during our study, a high phytoplanktonic biomass was determined in the water of the pond in the afternoon, possibly due to solar radiation stress relief, nutrient availability, and the potential effect of wind mixing facilitating sediment–water exchange, which could also be evidenced by changes in the microbial community of both compartments (see next section).

Evidence of intense nutrient recycling changes between the morning and afternoon was inferred from our endpoint rate estimations (Table S2) with shifts associated with nitrification and other processes indicating the sensitivity of microbial community activities to environmental conditions in the peatland pond. The ammonia oxidation rate determinations using inhibitors presented values falling in the range of previous reports in the study area using tracers [16] and evidenced a higher ammonia oxidation rate during the morning compared with the afternoon. The influence of both photo-inhibition or low nutrient availability possibly due to phytoplankton competition could account for the low ammonia oxidation rates determined in the afternoon as in other aquatic environments [5,8]. Endpoint experiments indicated a high nutrient consumption during the morning evidenced by high nitrate and phosphate consumption (Table S2). Inhibitors used to target nitrifying microorganisms were effective for ammonia oxidation; however, other processes non-sensitive to azide were detected in our samples, especially in the afternoon since our experiments account for nitrite consumption and nitrate accumulation.

The diel patterns of greenhouse gas concentrations observed in our study, particularly the supersaturation of CH_4_ and CO_2_ in the afternoon and persistent N_2_O undersaturation, were consistent with active biogeochemical cycling modulated by microbial functional groups. Taxonomic data revealed the presence of methanogenic archaea such as Methanobacteria in the sediments (Figure S12), and taxonomic-based predictions using FAPROTAX indicated enrichment of fermentation and other anaerobic pathways in the afternoon samples (Figures S9 and S10), supporting microbial contributions to CH_4_ accumulation (Figure 3 and Figure S4), in line with previous observations in Salar de Huasco and warming experiments in freshwater wetlands [12,13]. Similar microbial phyla and predicted functions have also been reported in hyper-arid desert soils, where microbial communities adapt to solar radiation and oligotrophy through enriched pathways such as membrane transport and carbon metabolism [50]. This taxonomic and functional convergence suggests shared adaptive strategies across distinct extreme environments [51,52]. This diel CH_4_ increase may also reflect the positive feedback between temperature and microbial metabolism such as methanogenesis and respiration [53,54,55].

In contrast, the depletion of N_2_O, despite the presence of nitrifiers (Nitrosomonas and Nitrosopumilaceae), suggests a combination of low production and potential microbial consumption via denitrification, supported by predicted denitrifier functions in water and sediment communities (Figures S9 and S10) [4]. While a previous study from our group reported a negative correlation between viral-like particles (VLP) and N_2_O concentrations [15], we now interpret this connection with greater caution. It may reflect indirect effects such as enhanced microbial turnover or remineralization via viral lysis rather than direct viral control of N_2_O fluxes [51].

### 4.2. Microbial Communities Compartmentalization and Shifts During the Day

A higher microbial community richness was found to inhabit the sediment compartment compared with the water, indicating that despite its shallowness, peatland ponds’ microbial specialization occurs as observed previously [14,18]. The microbial community of the pond was formed mainly by well-known prominent bacterial groups associated with Alpha- and Gammaproteobacteria, Actinobacteriota, and Bacteroidota in the water and mostly Bacteroidota and Verrucomicrobiota, in agreement with previous studies in high-altitude wetland ecosystems of the region [10,52]. A lower contribution of archaea was also observed in the pond associated with euryarchaeota and, to a lesser extent, other taxa including ammonia-oxidizing archaea (Figure 7 and Figure S10), which were rare but present in the sediments and water with variable contribution at different sampling times, in agreement with previous reports [13,52].

In general, the microbial community exhibited significant temporal shifts, with different microbial taxa dominating at varying times of the day, reflecting the complex interplay between environmental conditions and microbial functional dynamics at different spatial and temporal scales in this ecosystem [13,52]. Specific ASVs shifters were determined to be closely associated with changes in eukaryotic photosynthetic organisms based on a potential bloom in the afternoon evidenced by biochemical (pigment quantification), chloroplasts identified based on 16S rRNA, and functional prediction based on taxonomy. These results agree with previous reports indicating the role of eukaryotes as photosynthetic and non-photosynthetic in shaping microbial communities in this system [52].

In our study, highly diverse photoautotrophic microorganisms associated with different domains of life were found, including eukaryotic photosynthetic organisms and bacteria such as cyanobacteria able to carry out oxygenic photosynthetic metabolisms and six other bacteria phyla, including the versatile aerobic anoxygenic phototroph (AAP), which is a widespread bacterium in aquatic ecosystems including wetlands [56,57].

The diel enrichment of specific nitrifying taxa, such as Nitrosomonas in the sediments during the afternoon and Nitrosopumilaceae and Nitrososphaeraceae in the water throughout the day (Figure 7), coincided with marked shifts in nitrification rates (Figure 4B). In particular, Nitrosomonas dominance at 15:00 h followed by its disappearance by the next morning suggests a short-term activation of AOB populations followed by photo-inhibition, an effect previously observed in aquatic ecosystems exposed to high-UV radiation [5,58]. In parallel, the increase in Bacteroidota abundance in the water during the late afternoon (Figure 5 and Figure S8) occurred alongside declining *F_v_/F_m_* values and increased phaeopigments (Figure 2D), indicating a possible role in degrading organic matter from phototrophs undergoing photo-inhibition. These observations align with functional predictions based on taxonomy (Figure S9) and highlight a close temporal coupling between microbial community shifts and biogeochemical processes such as nitrification and organic matter remineralization [4,8,59].

In addition to nitrifiers and degraders, we also observed diel changes in Gammaproteobacteria, which showed a marked decline in relative abundance during the afternoon (Figure 5 and Figure S8). Genera such as Hydrogenophaga and Polynucleobacter (Figure S7), associated with aerobic heterotrophic metabolism and active carbon cycling in aquatic systems [59], were dominant in the morning but diminished at 17:30 h. This pattern may reflect sensitivity to high UVR and oxidative stress, consistent with known photo-inhibitory effects on aerobic bacteria in transparent waters [1,5,8]. Their decline coincided with lower CO_2_ concentrations (Figure 3 and Figure S4), suggesting a diel reduction in heterotrophic respiration. This interpretation is supported by functional predictions indicating a reduced contribution of chemoheterotrophy and carbon oxidation processes in the afternoon samples (Figures S9 and S10), reinforcing the close temporal coupling between microbial community shifts and ecosystem biogeochemical responses under poly-extreme solar radiation conditions [13].

Although functional prediction based on taxonomy alone should be considered with care based on the high microbial diversity found in extreme ecosystems, our findings based on well-known photo and chemolithoautotrophic microbial groups support shifts in the microbial functional potential of the peatland pond. The significant diel variations in photosynthetic efficiency and greenhouse gas concentrations highlight the sensitivity of aquatic ecosystems to diel changes in abiotic factors, particularly light and temperature [48]. The observed microbial community shifts suggest that temporal variations in environmental conditions can drive functional changes within key microbial functional groups, potentially impacting nutrient cycling and greenhouse gas emissions [58,59]. This knowledge is crucial for understanding the role of microbial communities in biogeochemical processes, especially in the context of climate change, where extreme weather events and altered light regimes may become more frequent [60].

This dynamic functional restructuring in response to diel stressors echoes findings from microbial ecosystem models, which show that stress intensifies both positive and negative interactions among microbial taxa, with implications for functional resilience and biogeochemical output under extreme conditions [9].

### 4.3. Peatland Pond Systems as Natural Laboratories to Identify Key Biogeochemical Budgets and Microbial Communities Changes

Our findings underscore the pivotal role of microbial communities in driving key biogeochemical cycles, including those underpinning greenhouse gas budgets such as CH_4_, a gas highly sensitive to warming in freshwater ecosystems [49]. In the studied peatland pond, daytime temperature rises were consistently linked to elevated CH_4_ concentrations, mirroring patterns reported for other freshwater systems [49]. The functional signals associated with methanogenesis and denitrification supported by both taxonomic data (e.g., Methanobacteria; Figure S12) and FAPROTAX predictions (Figures S9 and S10) underscore the biogeochemical plasticity of microbial communities in these systems. Nitrification and denitrification metabolisms, along with consistently low N_2_O concentrations, also reflect potential microbial N_2_O consumption pathways [4,50]. The function of specific groups in peatland areas could be further expanded based on the role of functional microbial groups associated with methane and ammonia oxidation, which could be involved in other biogenic volatile organic compounds derived from peatland plants such as isoprene [61].

Despite our study being focused on a single diel cycle in a selected pond, our results are supported by previous research in our study area [11,13] and by the significance of biogeochemical cycles in pond ecosystems worldwide. Peatland pond studies have been usually centered in high-latitude areas, and thus, our study highlights the importance of carrying further research efforts to put high-altitude wetlands and salar peatland ponds areas on the map of peatland research studies globally. However, further studies are needed to support the temporal and spatial replication and enhance generalizability of high-altitude peatland pond responses.

Given their high microbial diversity, rapid environmental response, and relevance to carbon and nitrogen cycling, peatland ponds emerge as biogeochemical hotspots with disproportionate contributions to climate-relevant gas fluxes [62,63]. Their dynamic microbial responses position them for detecting early signals of climate-driven changes in fragile Andean wetland landscapes [10,12,13]. This highlights the relevance of high-altitude peatland ponds as early-warning systems, complementing other climate change sentinel ecosystems such as Arctic permafrost, alpine lakes, and coral reefs [61,62].

## 5. Conclusions

This study demonstrates that peatland pond ecosystems are highly dynamic environments where abiotic factors and microbial communities interact closely, leading to significant diel variations in photosynthetic performance and greenhouse gas emissions. The observed shifts in microbial community composition and functional dynamics suggest that microbial populations are highly responsive to environmental changes, which could have important implications for ecosystem functioning and biogeochemical cycling. Understanding these interactions is crucial for predicting the impact of environmental changes on aquatic ecosystems, particularly in the context of global climate change. Therefore, locally and regionally, peatland freshwater bodies areas can be considered as climate change sentinels.

## Data Availability

The original contributions presented in this study are included in the article. Further inquiries can be directed to the corresponding author.

## Supplementary Materials

The following supporting information can be downloaded at https://www.mdpi.com/article/10.3390/microorganisms13091990/s1. Figure S1: Map of the study; Figure S2: Photographs showing general pond sampling at dawn area; Figure S3: Photographs showing complete area of the pond sampled; Figure S4: Greenhouse gases concentration determined in the floating chamber; Figure S5: Gases and nutrient concentration changes; Figure S6: Principal coordinate analyses (PCoA) showing microbial community structure variability, Figure S7: Microbial community heatmaps; Figure S8: Linear discriminant analysis (LDA) plot of microbial ASV; Figure S9: Functional prediction analysis in the water, based on taxonomy; Figure S10: Functional prediction analysis in the sediment, based on taxonomy; Figure S11: Archaeal community diversity at a family level changes in the water (A) and sediments (B); Figure S12: Archaeal community diversity at a phylum level changes in the water (A) and sediments (B); Table S1: Dissolved GHG, phosphate, and silicic acid concentrations determined in the water pond during a daily cycle; Table S2: Nutrient rates of change (μM h^−1^) based on endpoint experiments using treatments to potentially differentiate nitrifying community contribution; Table S3: Spearman correlation rank analyses results highlighting significance at *p* < 0.05. In green are positive significant correlations, and in red are negative significant correlations; Table S4: Alphadiversity of the planktonic and benthic microbial community based on richness, diversity, and evenness indexes; Table S5: Anoxygenic phototrophic bacteria and their classification in higher taxa.
