# Supplementary material for: Peatland Pond Microbiome and Biogeochemical Responses to Solar Radiation Extremes in a High-Altitude Wetland, Salar de Huasco, Chile
Source: Microorganisms. 2025 Aug 26;13(9):1990. doi: 10.3390/microorganisms13091990 (PMC12472180; doi:10.3390/microorganisms13091990)
Supplement: Supplementary file 1 [file microorganisms-13-01990-s001.zip › Supplementary Figures.pdf]

Article

Peatland pond microbiome and biogeochemical responses to solar radiation extremes in a high-altitude wetland, Salar de Huasco, Chile.

Yoanna Eissler[1], Alfredo Yanez-Montalvo[2,3], Paula S.M. Celis-Plá[4,5], Marcela Cornejo-D'Ottone[6], Andrés Trabal[4,7], Cristina Dorador[8,9], Claudia Piccini[10], Luisa I. Falcón[2], Polette Aguilar-Muñoz[5,11] and Verónica Molina[5,11]*

Supplementary Materials: Figures

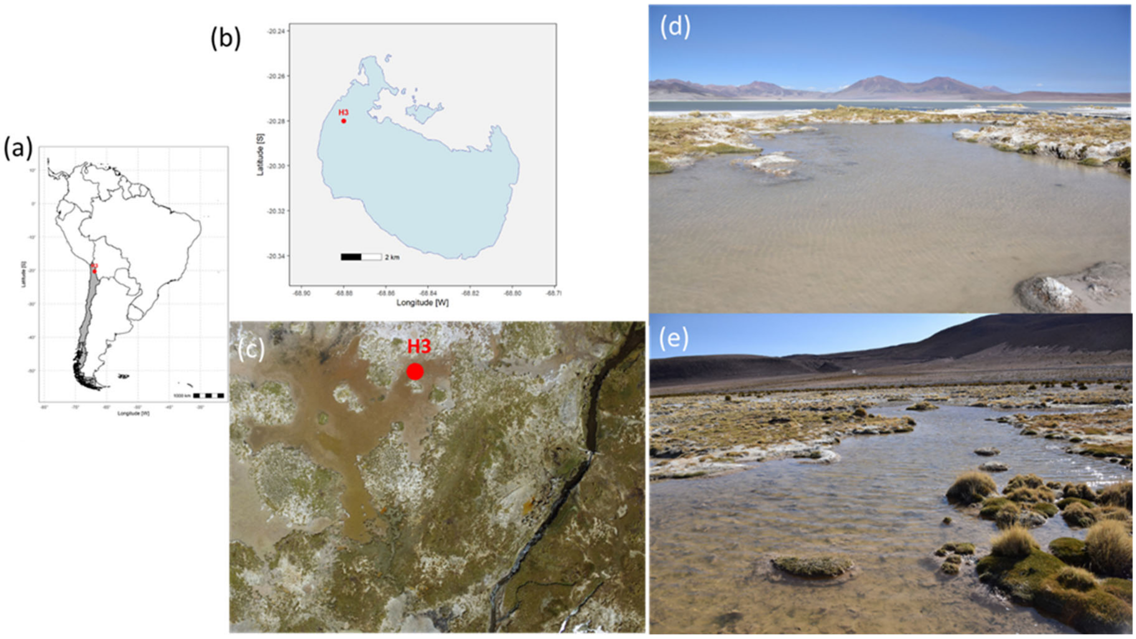

Figure S1. (a) Map of the study area with reference to South America, (b) Salar de HUasco and sample site H3 (c) DRON photograph of the studied pond and H3 site, (d, e) pictures of the pond at the sampling site.

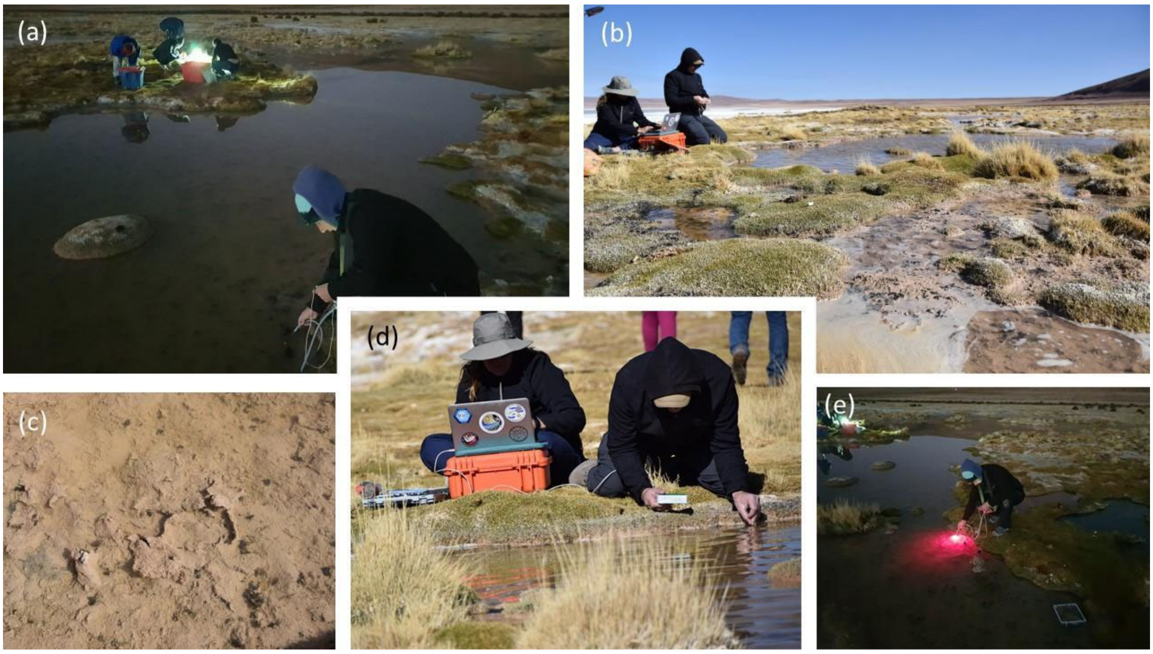

Figure S2. Photographs showing (a) general pond sampling at dawn, (b) measuring PAR radiation in the water column with a biospherical PAR sensor (US-SQS, Walz GmbH, Effeltrich, Germany) (c) unconsolidated microbial mat at the sediment surface (d) measuring PAR radiation just below the water surface and every 0.1 m depth in the water column with a biospherical PAR sensor (US-SQS, Walz GmbH, Effeltrich, Germany) (e) measuring of the *in vivo* chlorophyll-a fluorescence of the PSII with a Mini PAM II (Walz, GmbH, Effeltrich, Germany). Photos taken by Y. Eissler and P.S.M. Celis-Plá.

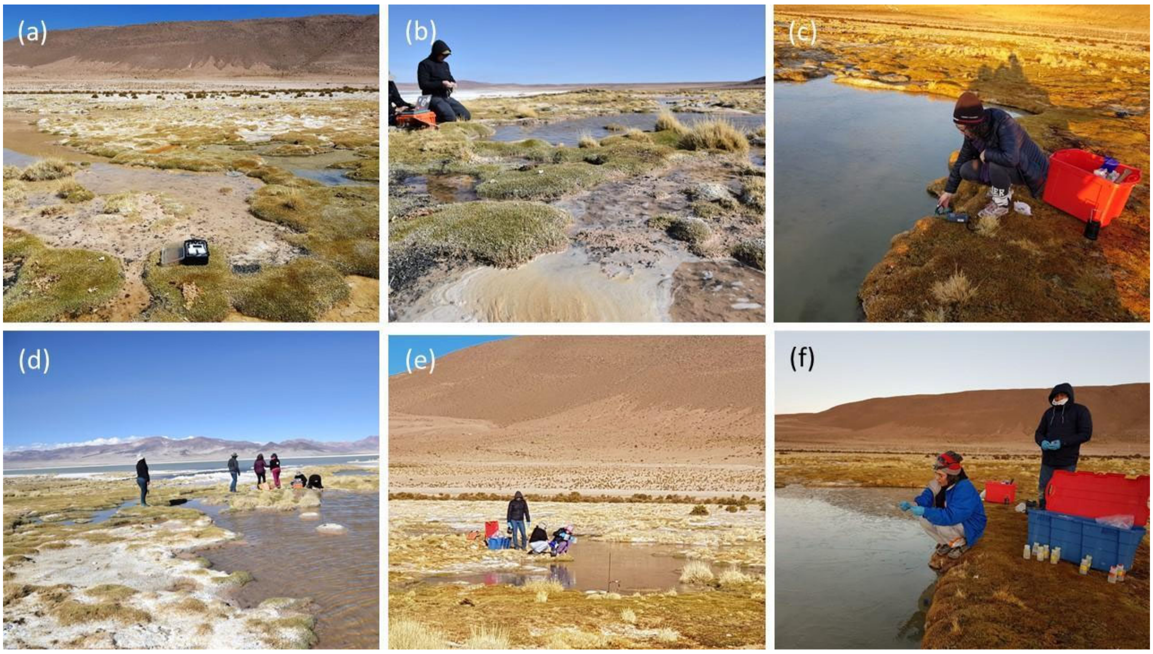

Figure S3. Photographs showing (a) complete area of the pond sampled and PAR and UVA-UVB Apogee sensors (Apogee Instruments, United States) with a data logger (HOBO UX120-006M, Hobo Data Loggers Australia), (b-f) general sampling procedure, (c) Multiparameter sampling for physicochemical variables and (f) setting for nitrogen uptake experiments. Photos taken by Y. Eissler and P.S.M. Celis-Plá.

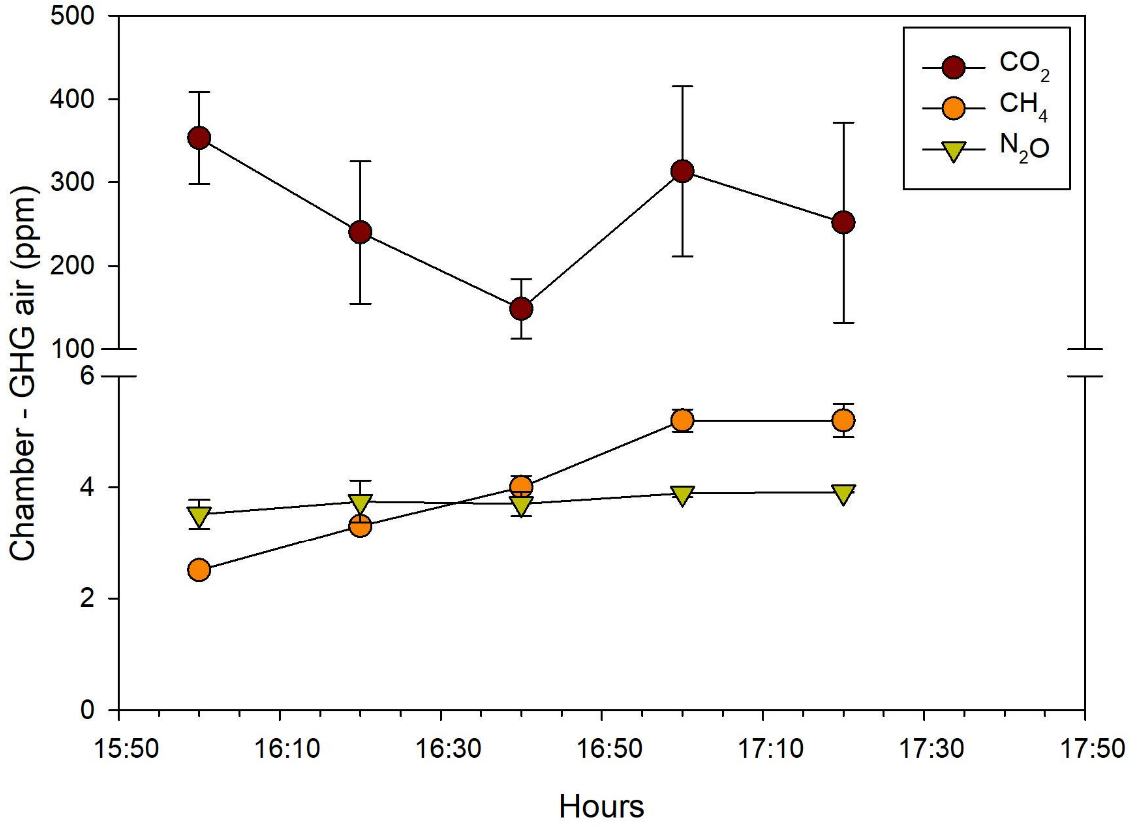

Figure S4. Greenhouse gases concentration determined in the floating chamber in the pond surface during the afternoon.

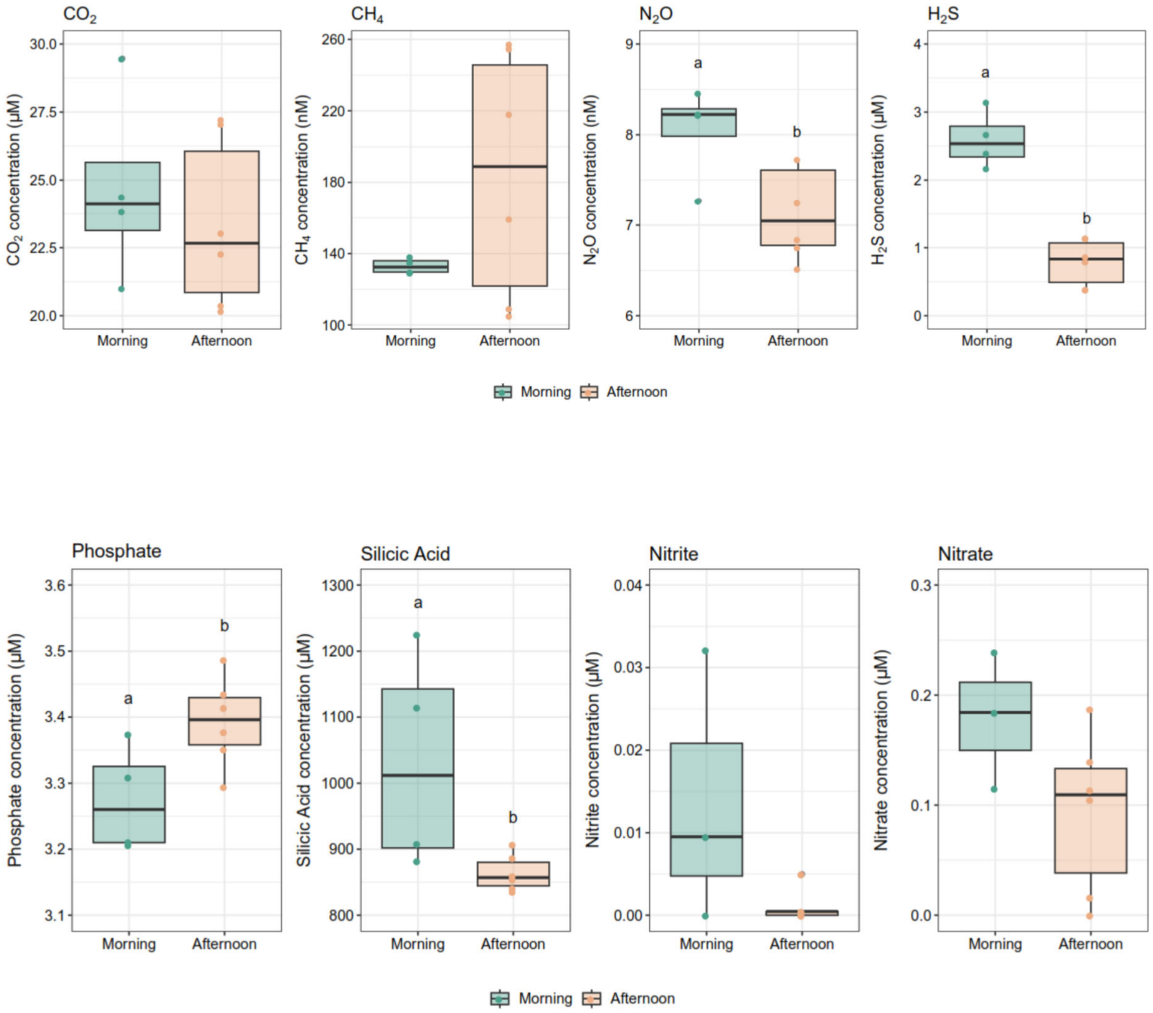

Figure S5. Gases and nutrient concentration changes compared between the morning and afternoon measurements tendencies. Significant differences were included considering $p<0.02$: $F=9.35$ (nitrous oxide), $F=56.86$ (sulfide), $F= 6.30$ (Phosphate) and $F=6.26$ (Silicic Acid).

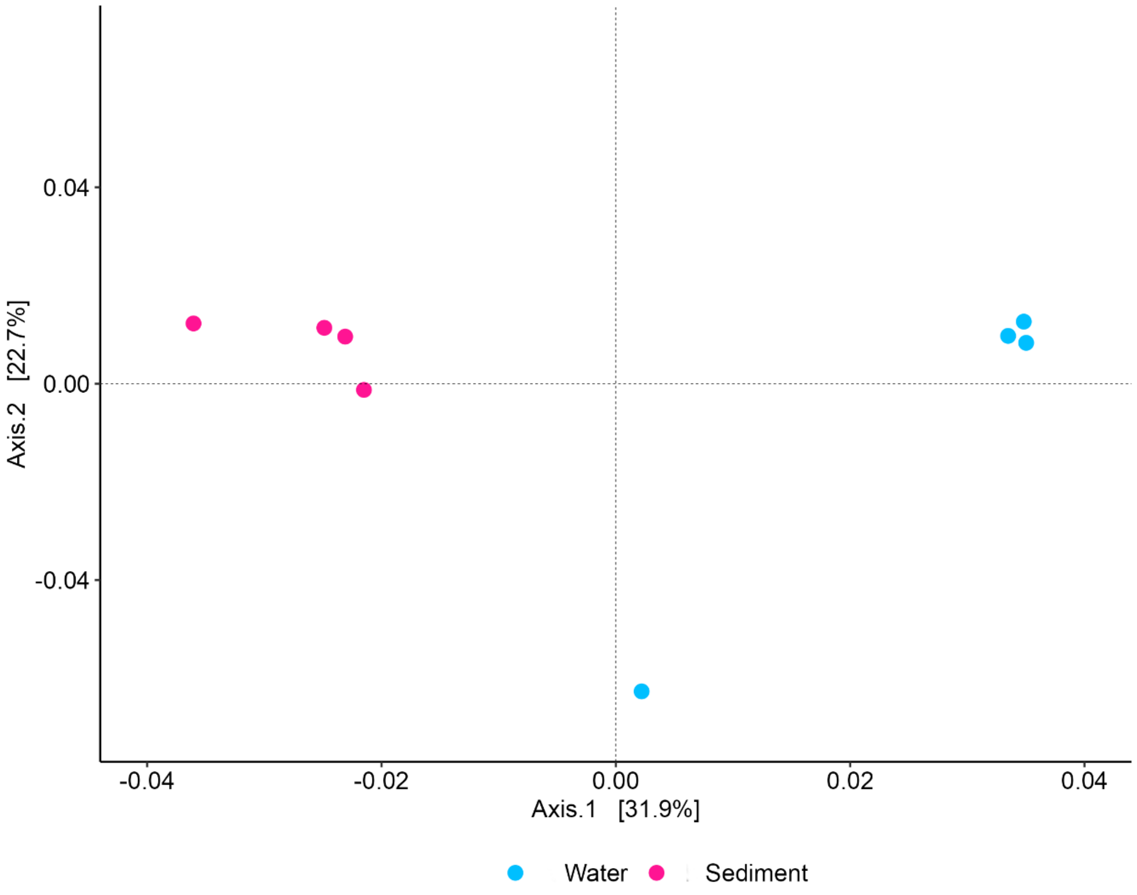

Figure S6. Principal Coordinate Analyses (PCoA) showing microbial community structure variability and discrimination between the sediment and water compartment.

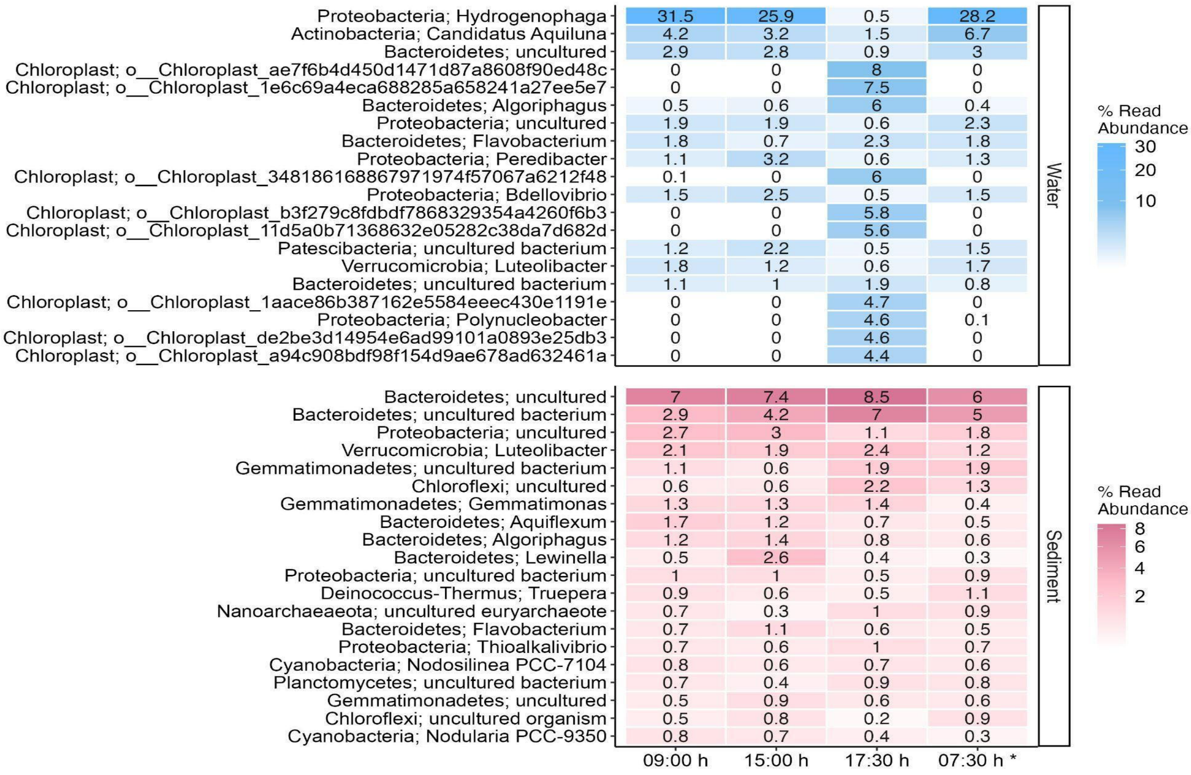

Figure S7. Microbial community heatmaps considering top 20 genera in the water (A) and sediments (B).

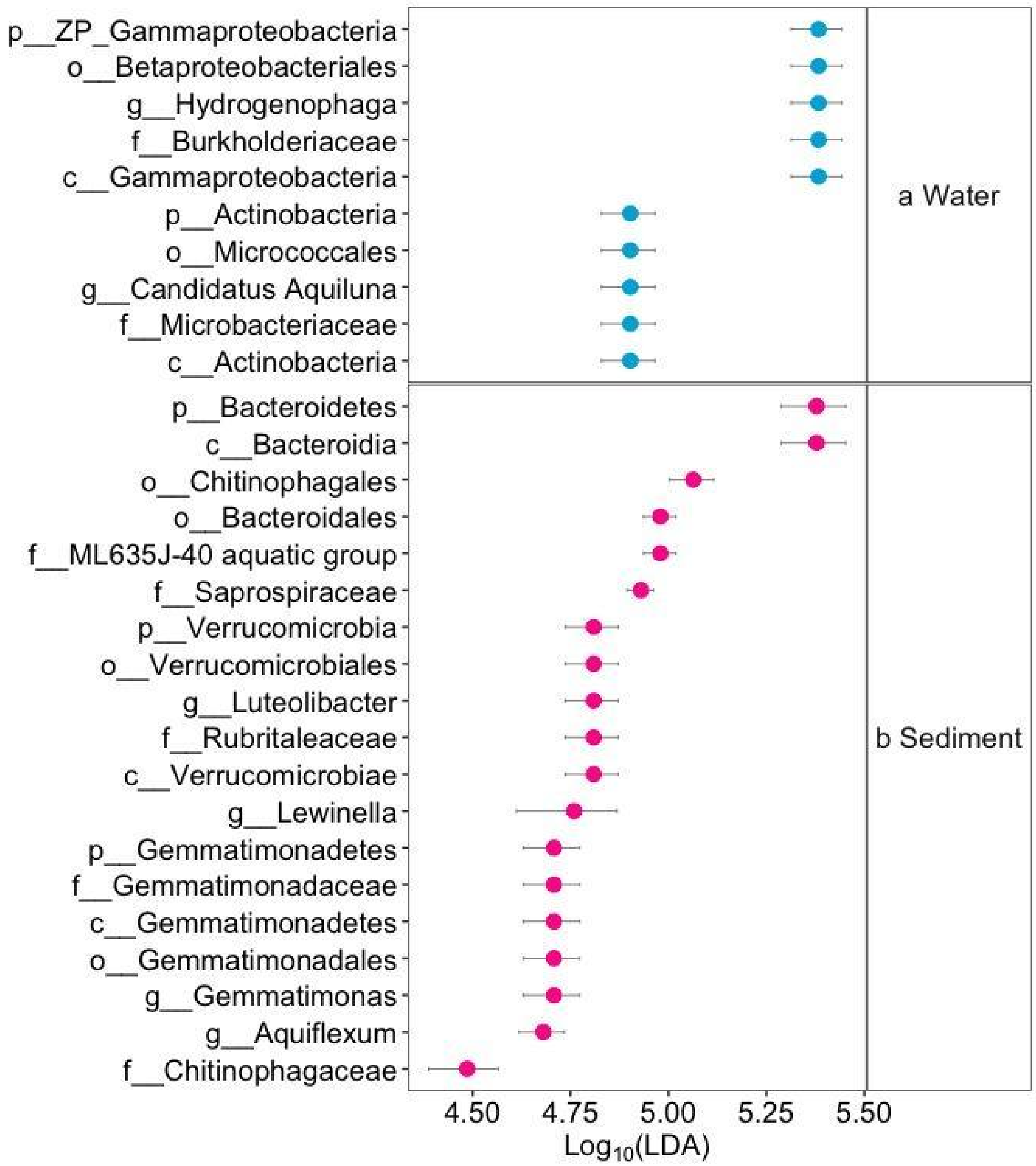

Figure S8. Linear Discriminant Analysis (LDA) plot of microbial ASV at the affiliated taxonomic level enriched in Afternoon and Morning considering a threshold LDA scores >2 (Wilcoxon test $p<0.05$).

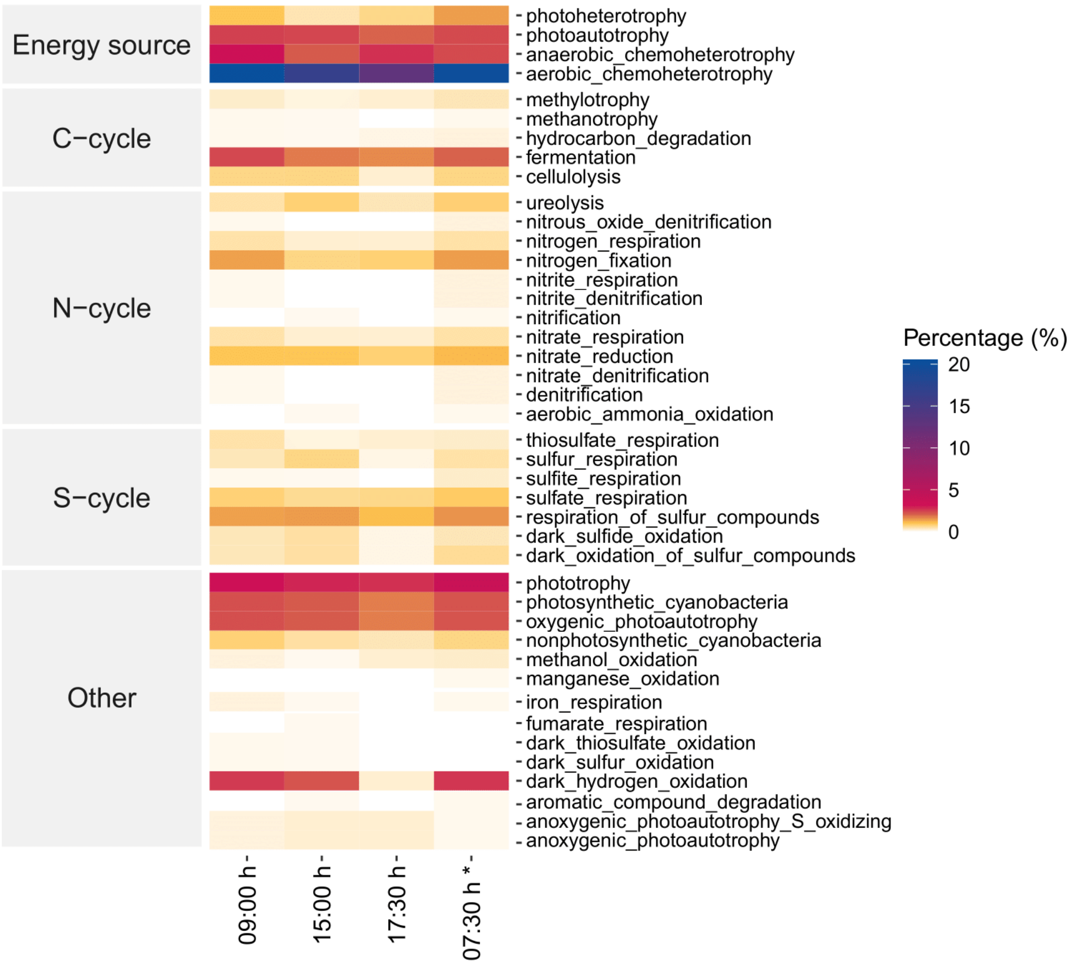

Figure S9. Functional prediction analysis in the water, based on taxonomy.

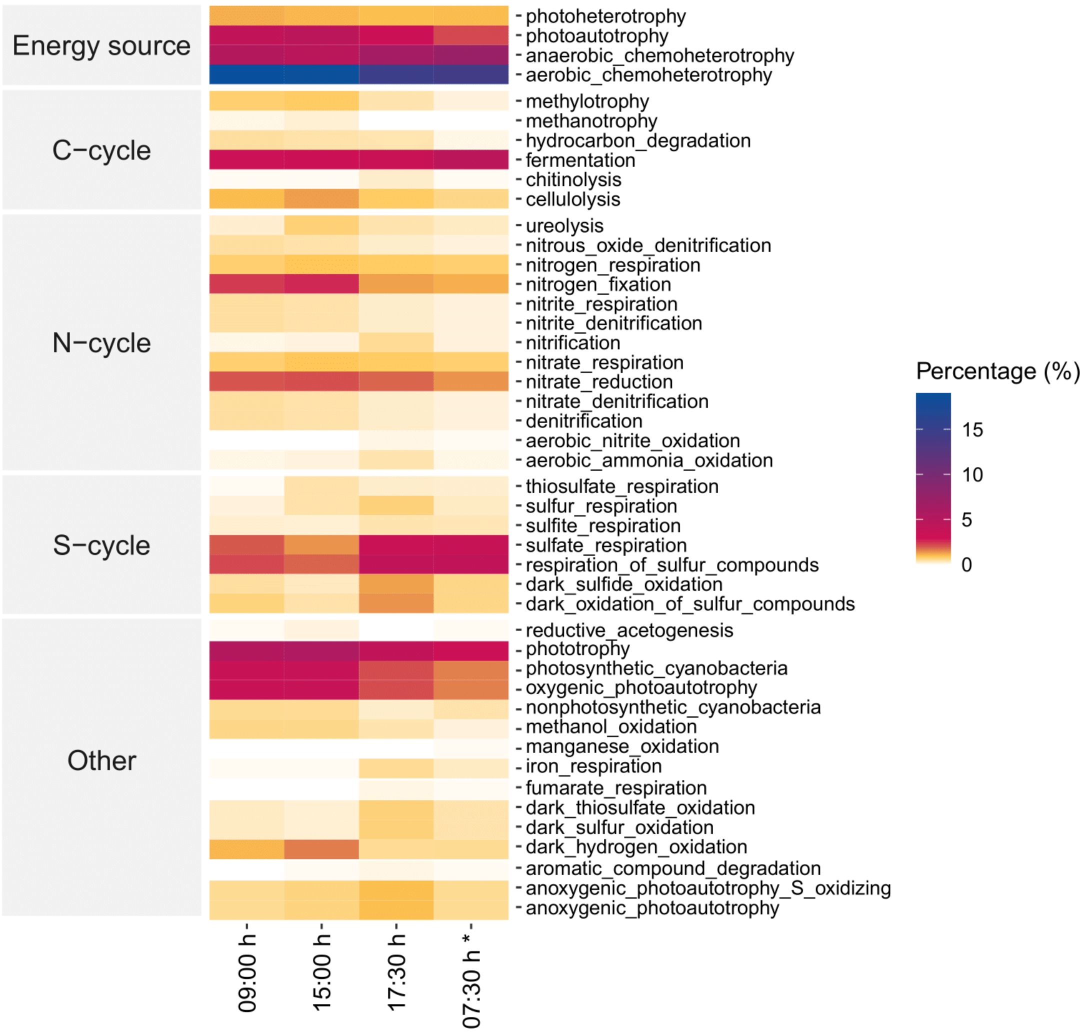

Figure S10. Functional prediction analysis in the sediment, based on taxonomy.

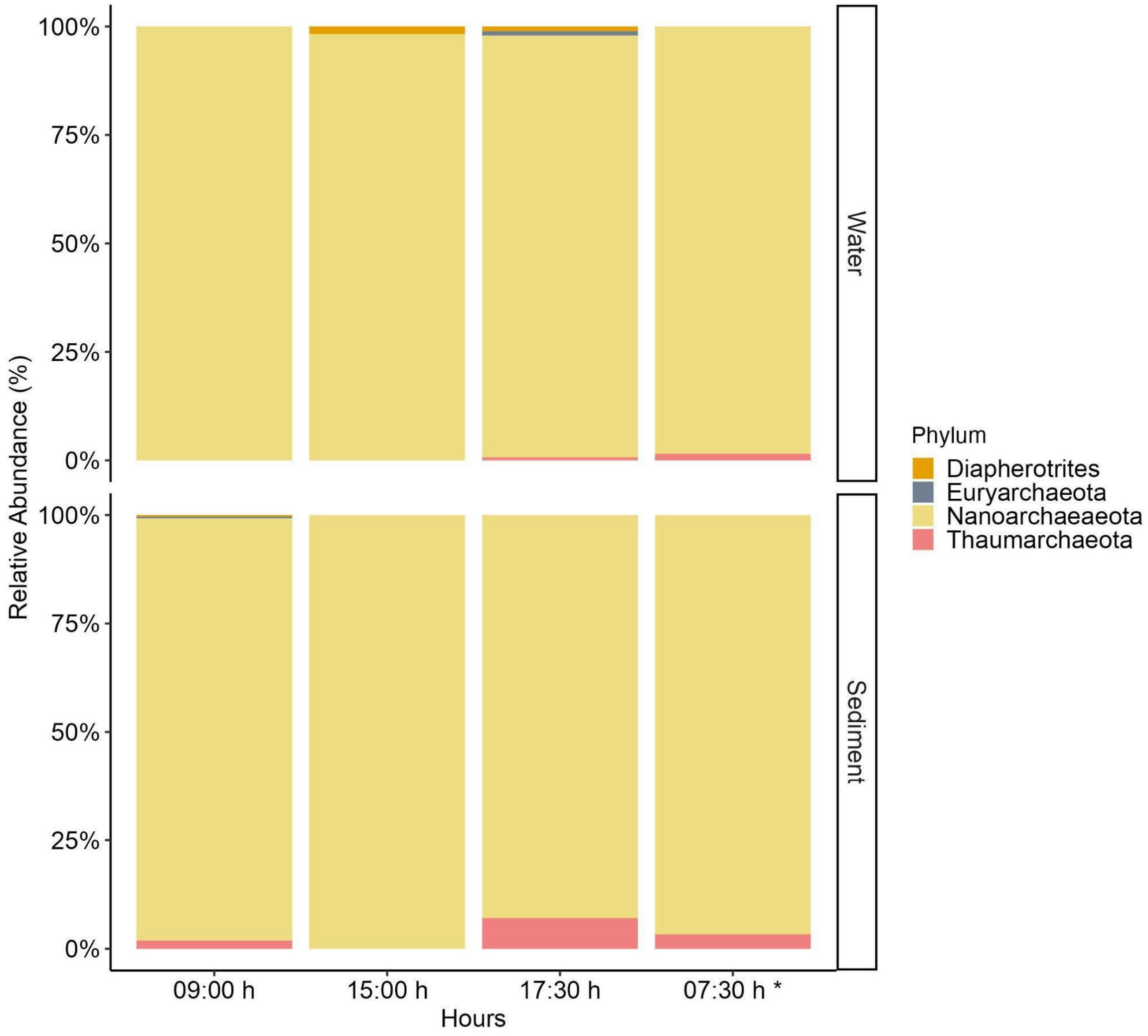

Figure S11. Archaeal community diversity at a phylum level changes in the water (A) and sediments (B).

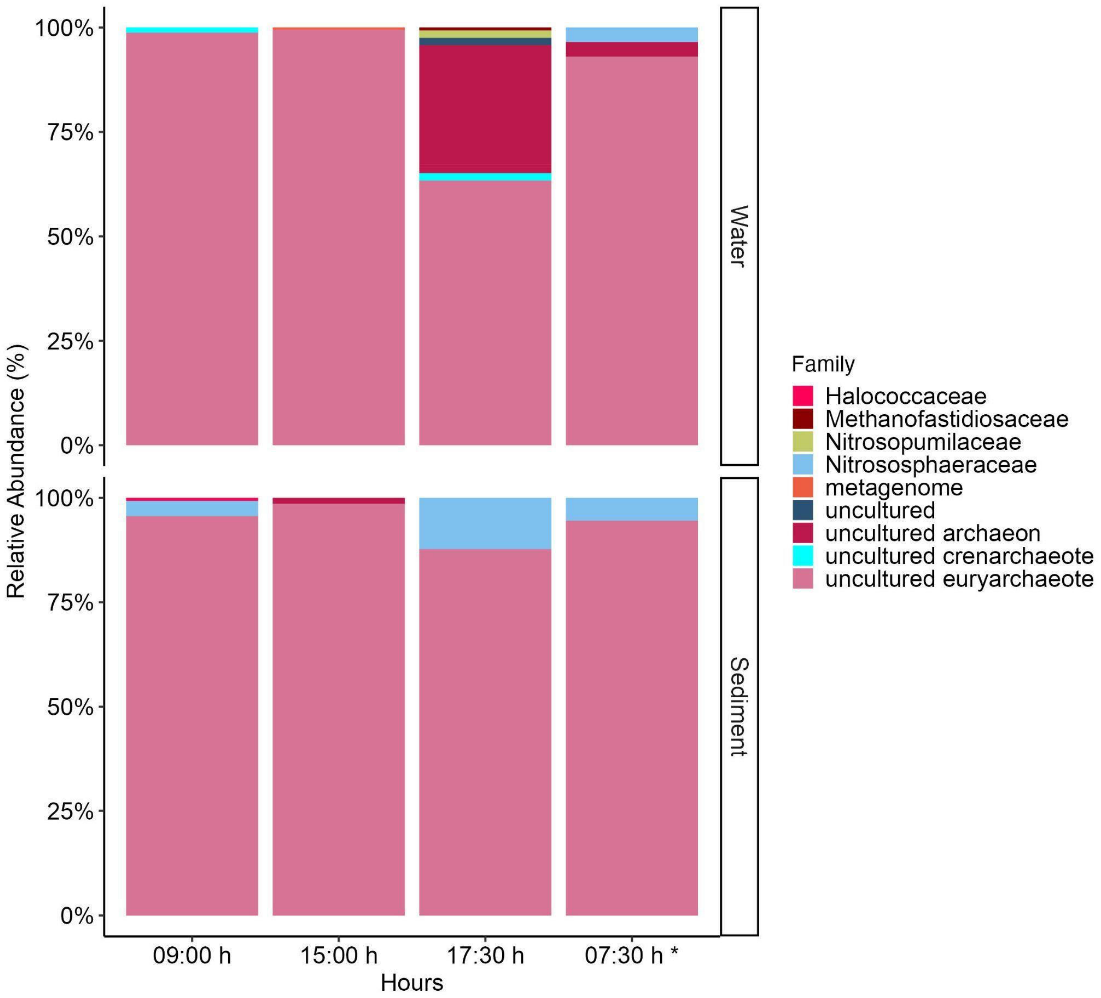

Figure S12. Archaeal community diversity at a family level changes in the water (A) and sediments (B).
